# A Randomized Comparison of In-hospital Rescuer Positions for Endotracheal Intubation in a Difficult Airway

**DOI:** 10.5811/westjem.2018.4.37227

**Published:** 2018-05-15

**Authors:** Joanna M. Le Parc, Jason J. Bischof, Andrew M. King, Sarah Greenberger, David P. Way, Ashish R. Panchal, Geoffrey I. Finnegan, Thomas E. Terndrup

**Affiliations:** * Immediate Health Associates, Westerville, Ohio; † The Ohio State University, Department of Emergency Medicine, Columbus, Ohio; ‡ University of Arkansas for Medical Sciences College of Medicine, Department of Emergency Medicine, Little Rock, Arkansas

## Abstract

**Introduction:**

Emergency endotracheal intubation (ETI) is a common and critical procedure performed in both prehospital and in-hospital settings. Studies of prehospital providers have demonstrated that rescuer position influences ETI outcomes. However, studies of in-hospital rescuer position for ETI are limited. While we adhere to strict standards for the administration of ETI, we posited that perhaps requiring in-hospital rescuers to stand for ETI is an obstacle to effectiveness. Our objective was to compare in-hospital emergency medicine (EM) trainees’ performance on ETI delivered from both the seated and standing positions.

**Methods:**

EM residents performed ETI on a difficult airway mannequin from both a seated and standing position. They were randomized to the position from which they performed ETI first. All ETIs were recorded and then scored using a modified version of the Airway Management Proficiency Checklist. Residents also rated the laryngeal view and the difficulty of the procedure. We analyzed comparisons between ETI positions with paired t-tests.

**Results:**

Forty-two of our 49 residents (85.7%) participated. Fifteen (35.7%) were female, and all three levels of training were represented. The average number of prior ETI experiences among our subjects was 44 (standard deviation=34). All scores related to ETI performance were statistically equivalent across the two positions (performance score, number of attempts, time to intubation success, and ratings of difficulty and laryngeal view). We also observed no differences across levels of training.

**Conclusion:**

The position of the in-hospital provider, whether seated or standing, had no effect on the provider’s ETI performance. Since environmental circumstances sometimes necessitate alternative positioning for effective ETI administration, our findings suggest that there may be value in training residents to perform ETI from both positions.

## INTRODUCTION

Airway management in general, and endotracheal intubation (ETI) more specifically, is an essential skill for both prehospital and in-hospital providers. The procedure is performed an estimated 265,000 times annually in United States (U.S.) emergency departments (ED).^1^ Accordingly, ETI is a core competency that Accreditation Council of Graduate Medical Education-accredited emergency medicine (EM) training programs are required to teach and assess.^2^ Currently, EM residency programs throughout the nation train their residents to perform ETI from a standing position. However, in the setting of the ED an emergency physician (EP) may be compelled to intubate at low bed heights to accommodate the simultaneous performance of high-quality chest compressions on a cardiac arrest patient.^3^ Furthermore, in the realm of emergency care, difficult environmental circumstances (such as mass casualty or disaster events) or difficult patient conditions may require rescuers to adapt their position for ETI.^4^ While conditions may dictate alternatives to standing for ETI, we have little evidence that EM residents can easily adapt from the standing position in which they are trained.

Studies of out-of-hospital providers (paramedics) have demonstrated that rescuer positions do not influence airway management results in the prehospital setting, especially those involving patients lying on the ground.^5^–^7^ One such study found no clinically relevant differences between paramedic positions for delivering ETI from the ground, which included prone, sitting, kneeling, and straddling-the-patient positions.^5^ The other study demonstrated that paramedics required fewer attempts when performing ETI from a left lateral decubitus position (relative to a patient lying supine on the ground), when compared to performing ETI from the kneeling position.^6^

In-hospital providers such as EPs are traditionally trained to perform ETI from the standing position. Since little is known about the topic of positioning for ETI involving in-hospital providers, we sought to determine whether performing ETI from the seated position might contribute to improved ETI performance. This question became more compelling when we considered that the performance of ETI from a seated position has the potential for easy implementation in the ED setting. Thus, the purpose of this study was to compare the ETI performance of EM trainees from both seated and standing positions. More specifically, we sought to determine how the traditional standing position with stretcher at mid-chest compared to the seated position with regard to ETI difficulty and laryngeal-inlet visualization. A finding of favorable or comparable performance metrics from the seated position would have important implications for training, particularly in situations where ETI is challenging.

## METHODS

### Population

This was a prospective, randomized, experimental cross-over design. The target population was EM residents from one EM residency program in the Midwestern U.S. Residents were approached during conference and asked to volunteer to participate in this study. We used stratified random sampling to assign resident volunteers to one of two groups. Stratification ensured that both groups had equal numbers of residents from each program year of training: first (postgraduate year [PGY]-1); second (PGY-2); and third (PGY-3). This study was reviewed and approved by our institutional review board.

**Population Health Research Capsule**

What do we already know about this issue?

In-hospital providers typically stand at the head of the stretcher with the patient’s head at mid-chest level when performing endotracheal intubation (ETI).

What was the research question?

Does performing ETI from a seated position compare favorably to performing ETI from the traditional standing position?

What was the major finding of the study?

EM residents performed equally well in both positions. Furthermore, there were no differences in time, view, or difficulty.

How does this improve population health?

When challenges to effective ETI involve environmental circumstances, assuming a seated position may prove to be an effective alternative.

### Materials

The experimental setup was comprised of two parts: a difficult airway model and an audiovisual recording system. The difficult airway model was designed and used for another study, but is briefly described here.^8^ The model was composed of a simulation mannequin (the Deluxe Difficult Airway Trainer, Laerdal Medical Corporation, Wappingers Falls, NY) strapped to a rigid backboard and placed on a stretcher. The difficulty of this simulator was enhanced with two additional features: 1) a rigid cervical collar (Laerdal Stifneck) to limit neck flexion and jaw movement; and 2) the inflation of the tongue to 60 mm Hg static pressure.^8^ The mannequin was modified to include a pressure gauge allowing for the accurate measurement of tongue inflation pressure throughout the performance assessment. Supplies required for intubation of the mannequin were provided, including a laryngoscope, an endotracheal tube (ETT), a stylet, and a bag-valve mask.

The audiovisual recording system was comprised of two moveable, bullet type-recording cameras and one additional view from a video laryngoscope. The three camera views increased the likelihood that essential video images of the resident’s ETI performance were captured in a systematic fashion. The two bullet cameras were mounted perpendicular to, but above, the mannequin to allow for visualization of the resident and the mannequin in both a horizontal and vertical plane. The horizontal-view camera captured the resident’s body position and the force applied to the mannequin during intubation. The vertical-view camera monitored the resident’s hand positioning during procedures such as ventilation and lateral movement during intubation. The third camera view was from C-MAC direct laryngoscope device (video laryngoscope), which captured the view of the resident’s manipulation of the ETT. While the C-MAC can be used to help the provider visualize the path of the ETT, we did not allow residents to use the visualization screen during these exercises. Sound was captured by a separate microphone placed in the simulation environment. All three video feeds and the audio feed were processed through a digital video recorder that produced a synchronized recording of all three views on one screen in real time.

### Measurement Instrument

The Airway Management Proficiency Checklist (AMPC) is a 40-item instrument designed for measuring comprehensive airway management performance in prehospital providers (paramedics).^9^ The instrument is comprised of three subscales including intubation, ventilation, and back-up airway. We adapted the instrument for assessing in-hospital providers in the ED setting by using only items from the intubation subscale and then selecting from those only the items relevant to in-hospital providers. This reduced the AMPC to 12 items, which represented the most important tasks required by in-hospital providers for successful intubation.

### Performance Assessment

One group was assigned to perform an ETI on the difficult airway mannequin from the seated position first. For this encounter, the height of the stretcher was set at 61 cm (two feet) from the ground, which appeared comfortable for a typical provider. The second group was assigned to perform an ETI on the difficult airway mannequin from the traditional standing position first. For the standing encounter, the stretcher was set at mid-chest height of the provider. After completion of the first encounter, residents switched to the alternative encounter so that each resident performed an ETI from both the seated and standing position.

Following informed consent, the EM residents entered an in situ simulation environment resembling an ED treatment bay. They were provided with a brief but detailed patient scenario and were then asked to perform an ETI on the difficult airway mannequin, in either the seated or standing situation. All ETI encounters were recorded and stored for future evaluation by two EM faculty who have had significant airway- management expertise. After each encounter, residents were asked to rate the difficulty of the ETI encounter using a 10-point visual analogue scale (VAS) in which “0” was considered extremely easy, and “10” was considered extremely difficult.^10^ Additionally, residents were asked to rate their view of the glottis using the Cormack-Lehane classification system.^11^

### Scoring

Evaluators used the modified AMPC to assess the recorded performances of resident ETIs. To check inter-rater reliability, 22 of the 42 subjects (52%) were assessed by both evaluators. Evaluators scored each performance task using a dichotomous scale in which a “0” indicated that the task was either not correctly performed or not performed at all; or a “1” indicated that the task was correctly performed. Summary scores were generated for each performance assessment, seated and standing, by summing the number of “1s” and converting the sum to a percentage out of 12 (total number of items). For the 22 subjects assessed by both evaluators, we used an average of the two evaluators’ summary scores. When a resident required more than one ETI attempt, the evaluators were instructed to score only the successful attempt. The amount of time to successful ETI, defined as the time it took (in seconds) for the resident to place the ETT in the mouth and successfully pass the ETT through the vocal cords, was obtained from the digital recordings. For residents with multiple attempts, time was calculated cumulatively by summing their time across attempts.

### Data Analysis

We used paired (or dependent) t-tests to compare the seated and standing conditions for each of the following variables: the performance scores of residents; time to successfully passing the tube; resident ratings of difficulty; and the Cormack-Lehane classification of the view of the glottis. We used a Bonferroni adjustment for multiple comparisons, setting the critical value for alpha to .05/5=.01.^12^ We also used box and whisker plots to compare the residents by level of training on their ETI performance and their time to success in both the standing and seated positions. We assessed inter-rater reliability between the two evaluators on each of the performance items using percentage of agreement and Krippendorff’s alpha.^13^

## RESULTS

Forty-two of 49 EM residents (85.7%) from our program volunteered to participate in the study. Of the 42 participants, 15 (36%) were women. Slightly more PGY-1s (18 of 42, or 43%) participated than PGY-2s (11 of 42, or 26%) or PGY-3s (13 of 42, or 31%) (Table 1).

**Figure 1:**
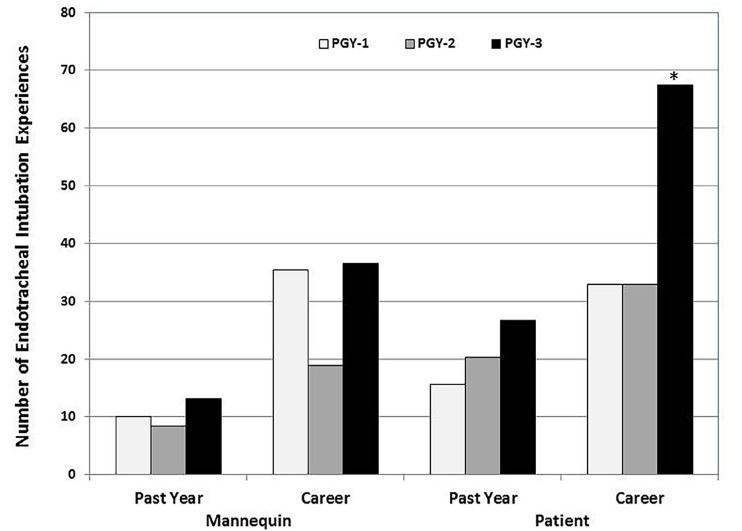
Residents’ experience with endotracheal intubation in the preceding year and over their careers in both simulated and actual patient care environments, by training level. *PGY*, postgraduate year. *PGY 3s had significantly more patient intubations over their career than did PGY 1a or 2s. (F=5.6, df=2, P≤.01).

Residents across different levels of training reported similar numbers of ETI encounters over the previous 12 months in both simulation (mannequin ETIs) and with live patients. However, as one would expect, PGY-3 residents reported significantly more live-patient ETI encounters over their career then did their peers (Figure 1).

The evaluators’ agreement was relatively good for most items (11 of 12) during their assessment of the resident’s standing ETI performance. The exception was “Maintains control over ETT placement.” The evaluators’ average percentage agreement for the standing assessment was 87.5%. The evaluators’ agreement for assessment of the seated performance was relatively good for nine of 12 items. The exceptions included “Flips up epiglottis to expose larynx;” “Passes tube through cords with limited or no impingement;” and “Maintains control over ETT placement.” The average percentage agreement for the seated position was 83.7% (Table 2). The three items in which evaluator agreement was less than “good” share the common characteristic of involving a high inference, qualitative judgment (exposure, impingement, or control).

Table 3 shows the descriptive and inferential statistics for the study’s measurements. Residents scored an average of three percentage points higher on the seated ETI performance assessment than they did on the standing performance assessment. The difference was not significant, and the associated effect size was small. Furthermore, we observed no other differences between the two ETI positions with regard to the number of attempts, the time to ETI success, and ratings of difficulty and view of the glottis.

Seven of the 42 residents (16.7%) required more than one attempt at ETI; five for the standing position, and two for the seated. Five of the seven residents successfully passed the ETT in the second attempt. The other two required more than two attempts, both in the standing position.

**Figure 2:**
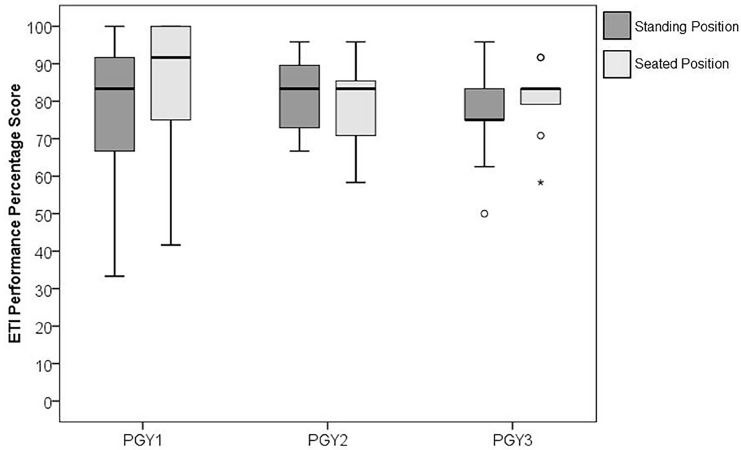
Box and whisker plots representing endotracheal intubation-performance scores by level of training for emergency medicine residents, in both the standing and sitting positions. *PGY*, postgraduate year.

**Figure 3:**
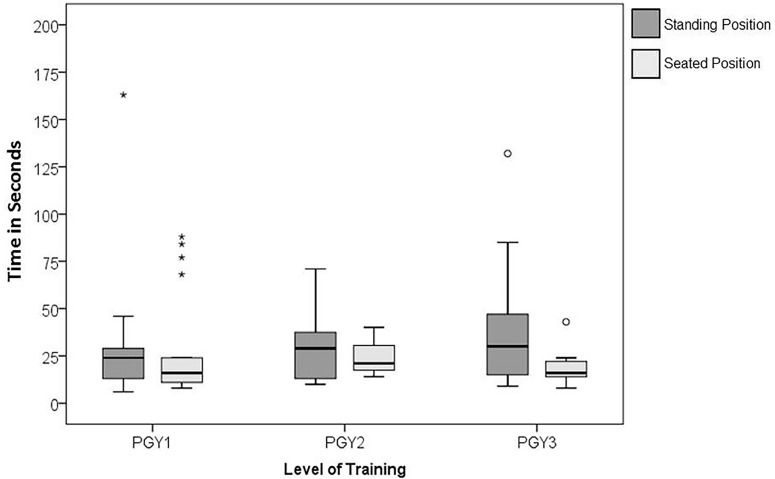
Box and whisker plots representing the distribution of time to intubation (in seconds) by level of training for emergency medicine residents, in both the standing and sitting positions. *PGY*, postgraduate year.

We observed that residents exhibited variability in their performance scores depending on their level of training, regardless of ETI position. PGY-1 scores were widely variable (as can be seen from the length of the box and whiskers in Figure 2) compared to PGY-2 scores, which were a little less variable, and then PGY-3 scores, which were much less variable. The box and whisker plot of the performance scores also shows that PGY-1s and 3s showed slightly better but not significantly better median performance in the seated position (Figure 2). Residents also demonstrated more variability in the time it took to successfully pass the tube from the standing position than from the seated position (Figure 3).

## DISCUSSION

In this study we evaluated whether the standing or seated position of EM residents impacted ETI performance or time to successful intubation. We expected that residents would perform better and pass the endotracheal tube more quickly from the standing position, since this is how nearly all residents are trained to perform ETI. We also expected that resident performance would improve as they progressed through their training.

We found that residents performed equally well in both the standing and seated positions. We also observed no differences in ratings of difficulty or laryngeal view between these positions. These findings are noteworthy since they suggest that there may be benefits to delivering ETI from a seated position. The change of position is easy to implement in many in-hospital settings, which makes these findings interesting for ED care. Further, since EPs, especially those involved in EMS and disaster/emergency preparedness, may find themselves needing to perform airway management in the field, learning to perform ETI from alternative positions may be important.

Another interesting observation was that when EM trainees were sitting, numerous residents chose to use their elbow as an anchor on the head of the bed just lateral to the patient’s head. This is a distinctly different approach to what residents are taught when performing ETI from a standing position and may indeed provide an advantage when performing ETI from a seated position.

Several studies have examined ETI positioning for in-hospital providers. One of the original studies demonstrated that minimum vs. maximum bed height (68.9 versus 101.3 cm) had no impact on time to intubation, success rate, or C-L view.^3^ Another study of in-hospital providers compared airway management performance across three different bed heights: at the knees, at mid-thigh height, and at the waist (anterior iliac spine height).^15^ These authors also found no difference across the three heights in terms of intubation time or outcome success; nor did they find differences in providers’ self-ratings of comfort, difficulty of the intubation, or the visual field. In both of these studies, the primary limitation was that they only measured outcomes and not the actual performance of the in-hospital providers during the ETI simulations.

We attempted to enhance these findings by incorporating actual performance measures into the study and found that performance was not affected by position. Finally, one large clinical evaluation of provider positioning for ETI involved a prehospital emergency medical unit in a suburb of Paris. In this study of 45 prehospital providers including EPs, residents, anesthesiologist and specialized nurses, there were no differences found in difficulty of tracheal intubation when comparing providers in the standing (referent) and kneeling positions (odds ratio [OR] [1.1]). However, this study did demonstrate an increased odds of difficulty in the lateral decubitus (OR [2.0]) and ventral decubitus positions (OR [2.0]).^4^

We chose the C-MAC device for this study because it could be used for both direct laryngoscopy and to provide a video record of the ETI performance for assessment.^16^ Participants were not permitted to see the video output from the C-MAC device. While video laryngoscopy is increasingly used for primary and secondary airway attempts, direct laryngoscopy remains a fundamental approach taught in virtually all programs providing instruction in airway management. Perhaps future research should investigate the effects of “types of laryngoscopy” combined with provider positioning on ETI outcomes. Knowing this, investigating the combined effects of various types of laryngoscopy and positioning on ETI may be worthwhile for future research.

## LIMITATIONS

Our observations were limited to one EM residency program in one institution. For more generalizable findings, this study will need to be replicated using a broader spectrum of in-hospital providers, i.e., EPs at more advanced levels of practice, and practitioners from other disciplines and institutions. We also failed to establish acceptable inter-rater reliability for at least three of the 12 items used to assess ETI performance. While we do not think that this affected our overall findings, it points to the need for either improved evaluator calibration/training or revision of these performance items to remove the high-inference qualifiers. Finally, we recognize that performance in simulated settings does not equal performance in the actual clinical setting, suggesting that further study in an actual clinical setting would be needed to confirm our findings.

## CONCLUSIONS

EM residents demonstrated equivalent ETI performance on a difficult airway model from both a standing and seated position. This was somewhat of a surprising finding, since residents in our program are trained to perform ETI from a standing position. We also found that while performance of PGY-1 residents was more variable, they scored at about the same level as their more experienced peers. All other comparisons, including time to placement of the ETT, laryngeal visualization, and number of attempts, were found to be comparable. Since environmental circumstances sometimes necessitate adaptation to a position other than standing for administering ETI, this study demonstrates that there may be value in training residents to perform ETI from both positions.

## Figures and Tables

**Table 1:** Numbers and percentages of emergency medicine residents by level of training, gender and participation in the sit-stand endotracheal intubation study.

	Participant		
	
Level	Female	Male	Both
1	8 (16)	10 (20)	18 (100)
2	2 (4)	9 (18)	11 (69)
3	5 (10)	8 (16)	13 (87)
Total	15 (31)	27 (55)	42 (86)

**Table 2:** Inter-rater reliability for version of the Airway Management Proficiency Checklist modified for in-hospital endotracheal intubation.

	Standing position		Seated position	
		
Performance task	% Agreement	K-Alpha	% Agreement	K-Alpha
Uses straight-to-cuff stylet curvature technique	100.0	NA	100.0	NA
Positions head properly	100.0	NA	100.0	NA
Grasps laryngoscope with left hand	95.5	.00	95.5	.00
Elevates mandible up and out w/laryngoscope	95.5	.00	95.5	.00
Flips up epiglottis to expose larynx	72.7	.47	68.2	.34
Inserts laryngoscope to appropriate depth	86.4	.73	81.8	.64
Moves blade tip smoothly without shaking or jerking	95.5	.83	81.8	−.08
Maintains view until ETT is at correct depth	95.5	.65	81.8	−.08
Passes ETT through cords with limited or no impingement	81.8	.25	68.2	−.16
Passes tube through cords in < 20 seconds	72.7	.46	68.2	.42
Maintains control over ET tube placement	54.5	−.02	63.6	−.19
Successfully intubates within 1 attempt	100.0	1.00	100.0	1.00

*K-alpha*, Krippendorff’s alpha; *ETT*, endotracheal tube; *ET*, endotracheal.

Notes: NA= When there is no variability in the rater’s scores (Both judges rated everyone the same) the K-Alpha cannot be computed due to invariant values, and the percentage agreement should be used instead. A K-Alpha=0 when both judges’ scores agree on all but 1 subject.^13^

**Table 3:** Comparison of 42 residents’ performances of endotracheal intubation from two positions

	Standing position		Seated position		T-test			
			
	Mean	SD	Mean	SD	t	df	p	es
Performance score (Pct)	78.2	14.8	81.2	13.5	1.2	41	.24	.213
N attempts	1.21	0.72	1.05	0.22	−1.4	41	.16	.323
Time in seconds	32.7	31.5	24.1	20.1	−1.6	41	.12	.331
Difficulty rating	4.17	2.68	4.16	2.36	−.03	41	.98	.004
Cormack-Lehane view rating	1.90	0.66	1.86	0.65	−.42	41	.68	.074

*ETI*, endotracheal intubation; *SD*, standard deviation; *t*, dependent t-test; *df*, degrees of freedom; *p*, probability value; *es*, effect size; *Pct*, percentage.

Notes: Bonferroni adjustment is alpha = .01. We computed the Cohen’s deffect size for correlated designs as recommended by Dunlop et al. (1996). All Cohen’s deffect sizes were interpreted as small or trivial.^14^
